# Successful Revascularization of Infrapopliteal Chronic Total Occlusions Using the Plantar Arch as a Conduit and Retrograde Pedal Access

**DOI:** 10.31486/toj.20.0085

**Published:** 2021

**Authors:** Tamunoinemi Bob-Manuel, Koyenum Obi, Zola N’Dandu

**Affiliations:** ^1^ Department of Cardiovascular Disease, John Ochsner Heart and Vascular Center, Ochsner Clinic Foundation, New Orleans, LA; ^2^ The University of Queensland Faculty of Medicine, Ochsner Clinical School, New Orleans, LA; ^3^ Department of Hospital Medicine, Ochsner Clinic Foundation, New Orleans, LA; ^4^ Department of Cardiology, Ochsner Clinic Foundation, Kenner, LA

**Keywords:** *Endovascular procedures*, *foot*, *ischemia*, *peripheral arterial disease*, *vascular access devices*, *vascular occlusion*, *wound healing*

## Abstract

**Background:** With the rising prevalence of critical limb ischemia (CLI), the pedal-plantar loop technique and retrograde access may be needed to increase interventional success.

**Case Report:** A 63-year-old female with severe peripheral artery disease presented with a 2-month nonhealing wound on the dorsum of her left foot despite wound care. We inserted a 65-cm Destination Guiding Sheath and crossed the right superficial femoral artery (SFA) chronic total occlusion (CTO) that we initially treated with a 4.0-mm Ultraverse balloon. We attempted unsuccessfully to cross the distal anterior tibial artery into the dorsalis pedis artery. We obtained antegrade access of the posterior tibial artery at the level of the ankle with a 2.9-French Cook pedal access kit. We inserted a 90-cm CXI catheter with a 0.014 Fielder XT wire and used the lateral plantar artery as a conduit to cross the dorsalis pedis artery and distal anterior tibial artery CTO with retrograde wire manipulation via lateral plantar artery. Finally, we performed distal anterior tibial and dorsalis pedis CTO balloon angioplasty with a 2.5 × 220-mm Ultraverse balloon and performed SFA percutaneous transluminal angioplasty and stenting with a 7.0 × 120-mm Zilver PTX stent, postdilated with a 6.0-mm Ultraverse balloon. We successfully established in-line flow to the foot with 3-vessel runoff. The patient's wound healed in a month.

**Conclusion:** Retrograde pedal access can improve the success rate of recanalization of below-the-knee disease in patients with CLI.

## INTRODUCTION

The prevalence of critical limb ischemia (CLI)—characterized by multilevel peripheral artery disease (PAD)—is increasing, and below-the-knee disease is especially difficult to treat.^1,2^ To avoid the major economic cost, disability, and mortality associated with CLI and amputation, an endovascular first approach is recommended.^3,4^

The pedal-plantar loop technique and retrograde access, together with an antegrade ipsilateral common femoral artery (CFA) approach, can increase the success rate of revascularization and the clinical outcome of below-the-knee disease, improving the wound-healing process.^5^ Tibiopedal access is increasingly being used as first-line approach because of increases in expertise, improved tools, and reduced complication rates in patients who are poor candidates for antegrade access.^6-8^

We present the case of a patient with a 2-month history of a nonhealing foot wound that responded to revascularization of the pedal arch.

## CASE REPORT

**Figure 1.**
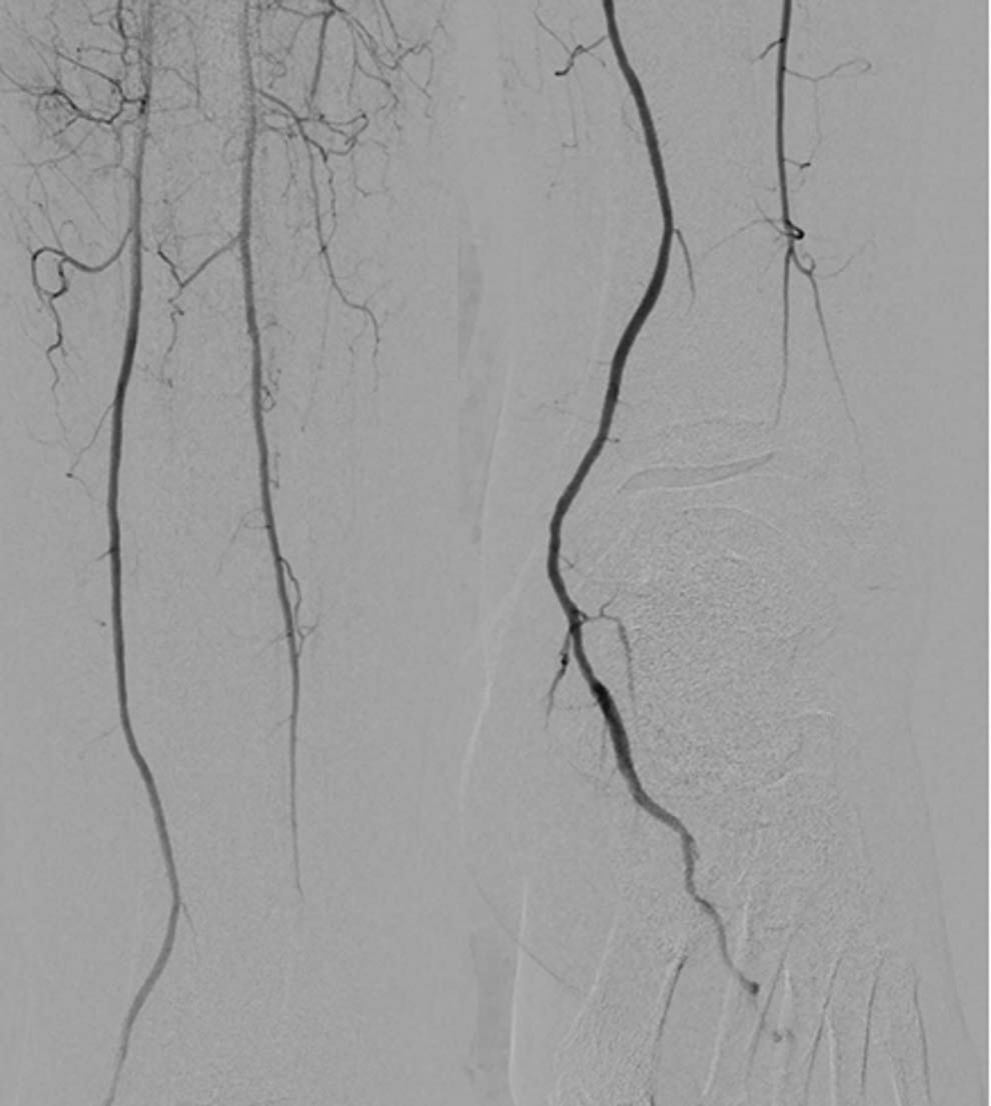
Preintervention angiogram of residual left superficial artery, distal anterior tibial artery, and dorsalis pedis artery chronic total occlusion.

A 63-year-old female with a history of coronary artery disease, hyperlipidemia, hypertension, borderline diabetes, obesity, and severe PAD (Rutherford grade V, Fontaine stage IV) was referred by podiatry for a 2-month nonhealing wound on the dorsum of her left foot despite aggressive wound care. The patient's procedural history included revascularization of the right superficial femoral artery (SFA) for CLI and chronic total occlusion (CTO) of residual left mid-SFA, distal anterior tibial artery, and dorsalis pedis artery (Figure 1).

Ideally, we would have obtained ipsilateral antegrade CFA access for adequate support, but obesity posed a high risk of bleeding. We obtained contralateral right CFA access using a micropuncture kit and then upsized to a 6-French (Fr) sheath. We administered heparin after the 6-Fr 65-cm Destination Guiding Sheath (Terumo Corporation) was exchanged. Next, we inserted a 135-cm NaviCross Support Catheter (Terumo Corporation) with a 0.035 Glidewire Advantage wire (Terumo Corporation) that crossed the SFA CTO. We initially treated the SFA CTO with a 4.0-mm Ultraverse balloon (BD). We telescoped an angled 150-cm CXI Support Catheter (Cook Medical) with a 0.014 Fielder XT wire (Asahi Intecc Co, Ltd) to cross the proximal segment of the anterior tibial artery CTO. We were not able to advance the catheter or the wire past the distal anterior tibial artery into the dorsalis pedis artery because of inadequate support and equipment length.

**Figure 2.**
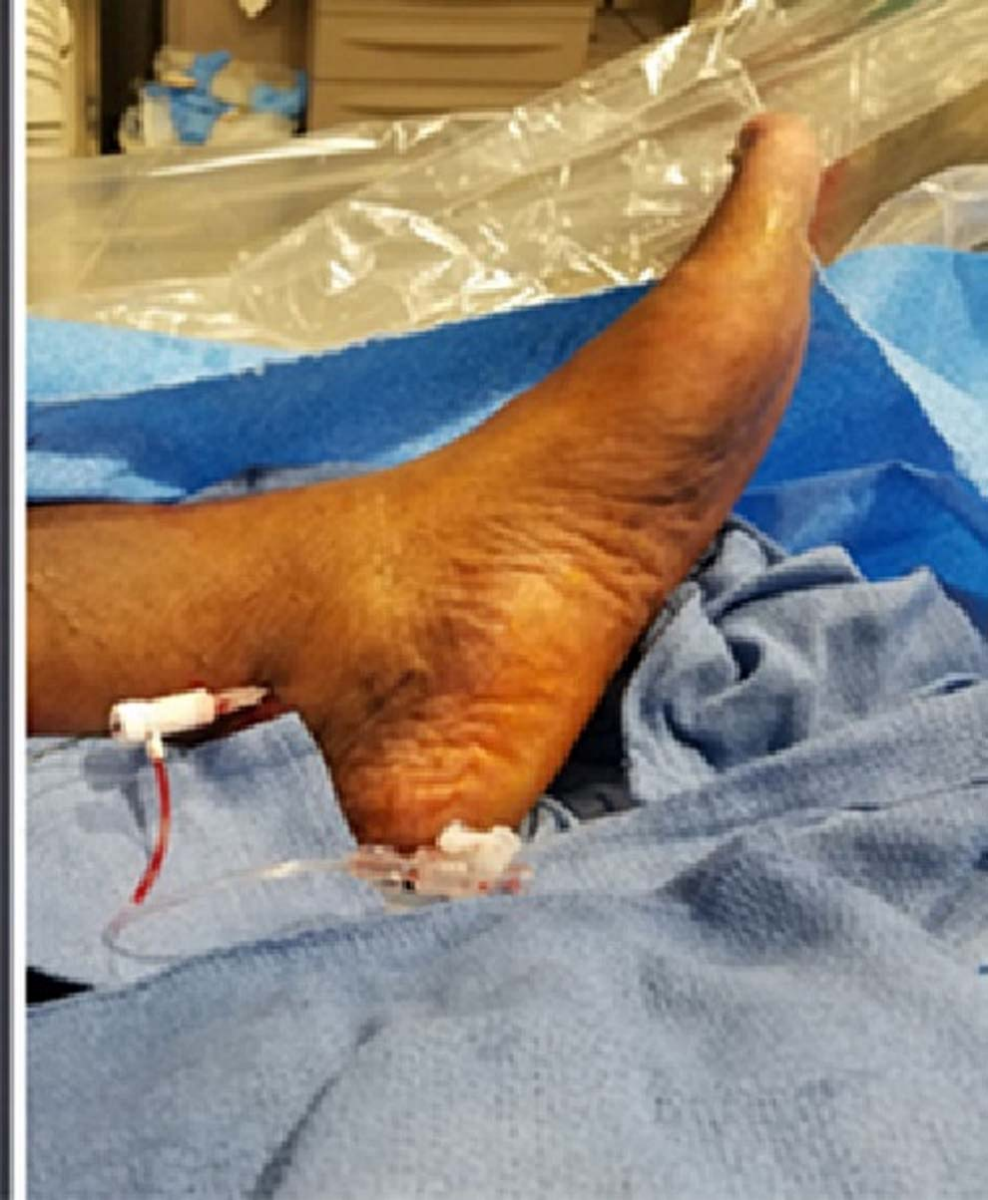
Antegrade access of the posterior tibial artery at the level of the ankle was obtained with 2.9-French Cook pedal access kit.

**Figure 3.**
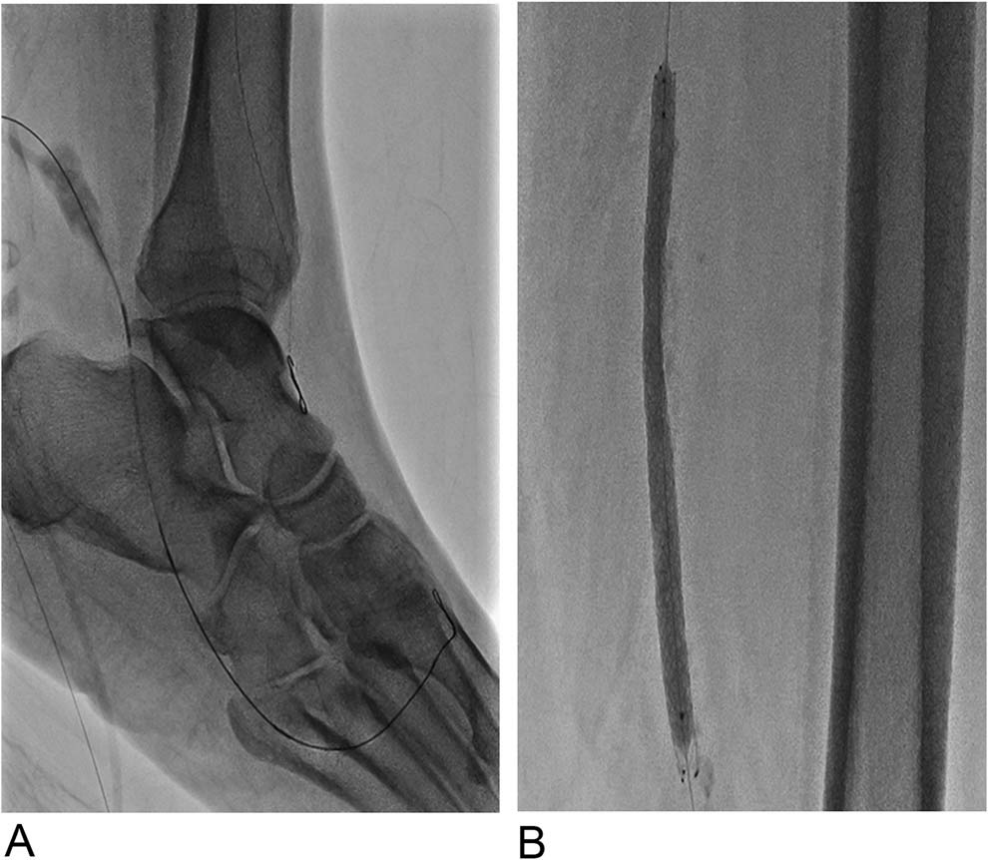
(A) Crossing the dorsalis pedis artery, and anterior tibial artery, chronic total occlusion with retrograde wire manipulation via the lateral plantar artery during the intervention. (B) Superficial femoral artery percutaneous transluminal angioplasty and stenting with 7.0 × 120-mm Zilver PTX stent during the intervention.

We obtained ultrasound-guided antegrade access of the posterior tibial artery at the level of the ankle with a 2.9-Fr Cook pedal access kit (Cook Medical) (Figure 2). We inserted a 90-cm CXI catheter (Cook Medical) with a 0.014 Fielder XT wire and used the lateral plantar artery as a conduit to cross the dorsalis pedis and distal anterior tibial CTO with retrograde wire manipulation (Figure 3A). Finally, we performed balloon angioplasty of the anterior tibial artery and dorsalis pedis artery with a 2.5 × 220-mm Ultraverse balloon and performed SFA percutaneous transluminal angioplasty and stenting with a 7.0 × 120-mm Zilver PTX stent (Cook Medical) postdilated with a 6.0-mm Ultraverse balloon at the end of the case (Figure 3B).

**Figure 4.**
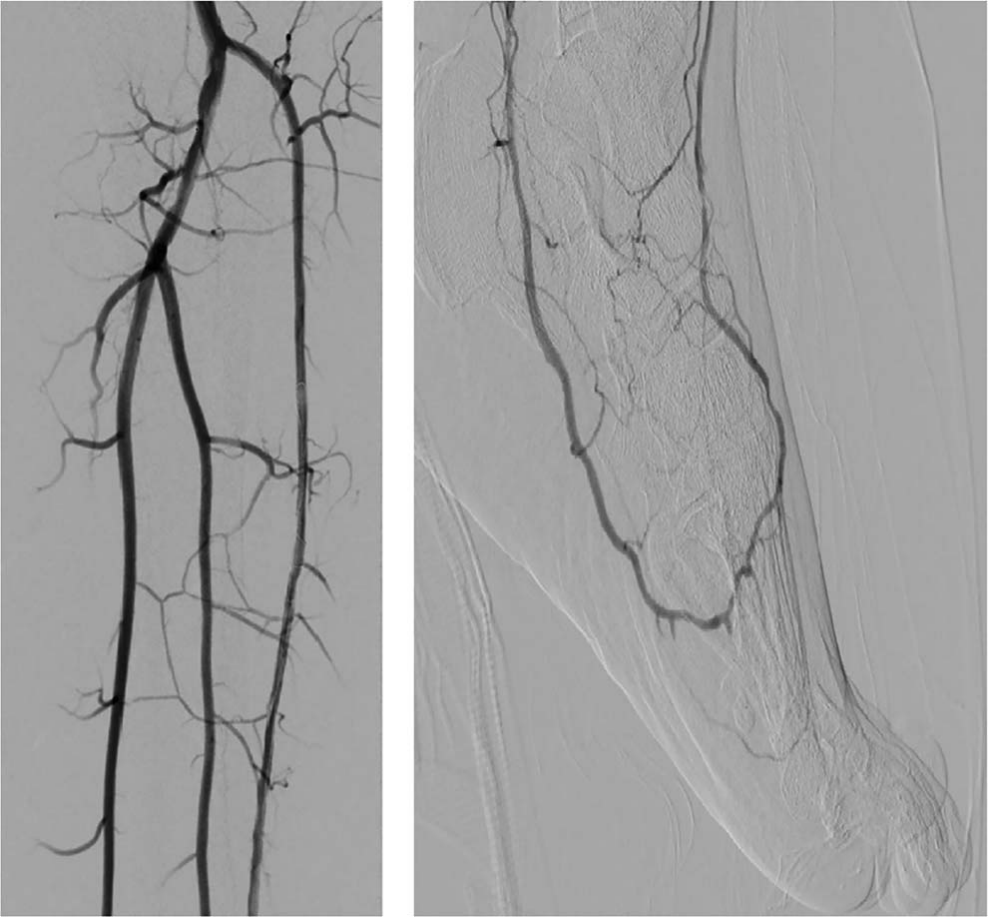
(A) Three-vessel runoff to the foot. (B) Patent pedal-plantar loop postintervention.

**Figure 5.**
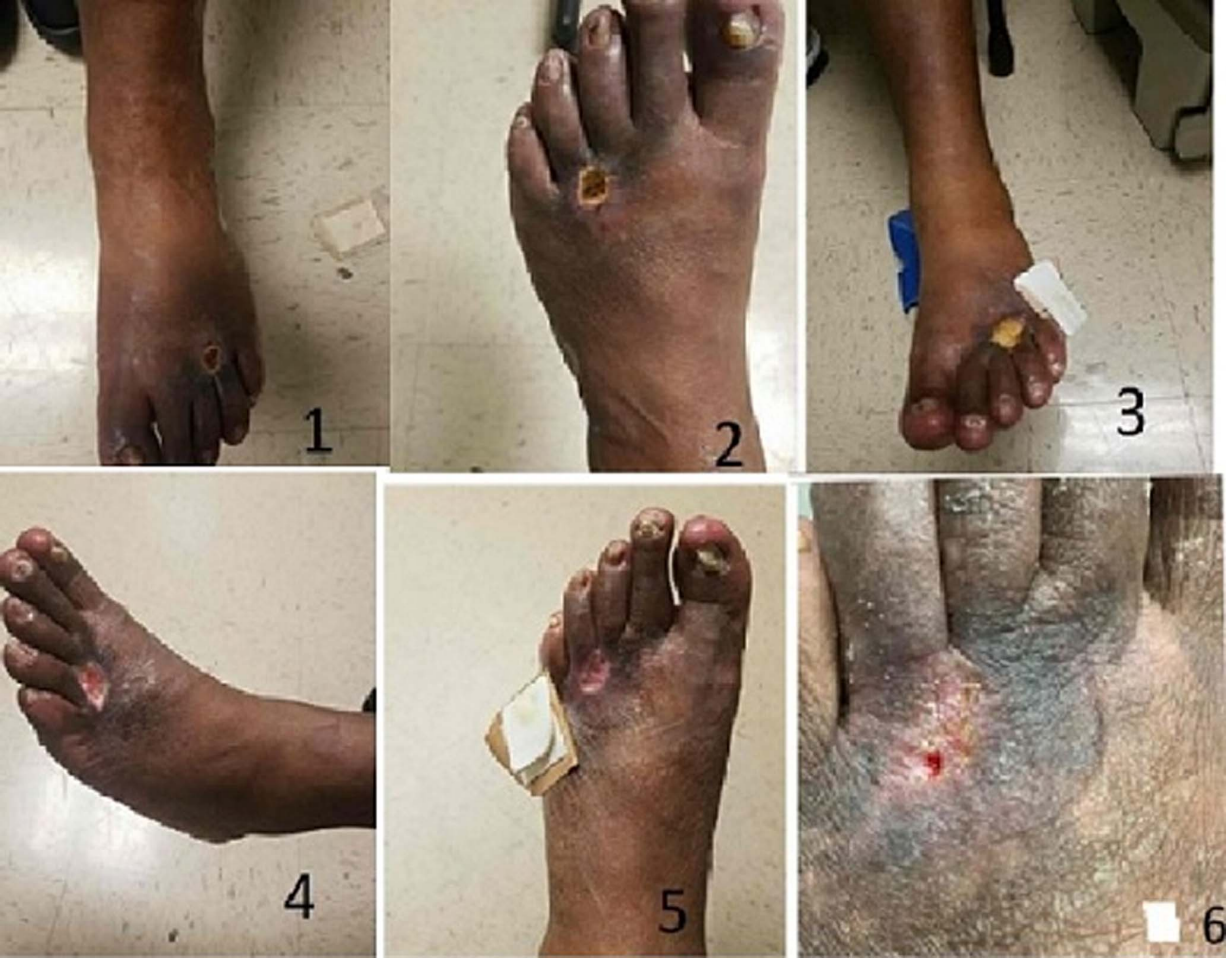
(1) Preintervention dorsal foot wound. (2-6) Wound recovery from 1 week postintervention to 1 month postintervention.

Postprocedural imaging showed a patent SFA with 3-vessel runoff (Figure 4). Right CFA access hemostasis was achieved with a Perclose ProGlide vascular closure device (Abbott Laboratories). Hemostasis of the posterior tibial access was obtained with manual pressure. The patient's wound healed in 1 month (Figure 5).

## DISCUSSION

Because of lack of awareness of PAD among patients and physicians and increasing comorbidities such as diabetes, the prevalence of CLI characterized by multilevel PAD is rising. More than 1.3% of patients with lower extremity PAD develop CLI, a severe clinical manifestation of PAD that is associated with an annual mortality rate of approximately 20%.^9^ The occurrence of CLI significantly worsens clinical outcomes in patients with PAD and adversely affects health care spending.^10^ Without proper intervention, CLI represents a considerable burden on the US health care system and individual morbidity and mortality and causes reduced health-related quality of life, particularly as the overall life expectancy increases.^11,12^

Advances in endovascular techniques, devices, adjuvant treatments, and operator experience have led to endovascular therapy being the preferred treatment of PAD and CLI.^4^ Use of tibiopedal access is increasing as a first-line approach because of increased expertise, improved tools, and reduced complication rates in patients who are poor candidates for antegrade access and for the treatment of patients with PAD suffering from claudication (Rutherford grades II and III) that has not yet progressed to CLI (Rutherford grades IV, V, and VI).^6,8^ In a retrospective study of 23 patients in 2014, 95% achieved a successful outcome—ability to cross lesion and achieve posttreatment stenosis <30%—using tibiopedal access with few complications.^11^

Tibiopedal access increases the success of staying within the true lumen, reduces overuse of costly CTO crossing tools and reentry tools, dramatically reduces fluoroscopy times, reduces bleeding risk, improves control because of proximity of the target lesion to the access, and reduces time to discharge.^13,14^ Very few complications have been reported in the literature, principally access-site thrombosis, which is usually salvaged and rarely needs surgery.^15,16^ Revascularization of the pedal arch has been shown to improve wound healing, especially in patients with diabetes.^17^

In addition to early revascularization, optimum medical therapy with a statin, antiplatelet therapy, and a multidisciplinary approach can improve outcomes. Procedurally, ultrasound-guided pedal loop access reduces vascular complications and radiation exposure to both patient and operator, compared to traditional access without ultrasound. Heparin is dosed using 80 U/kg with a goal activated clotting time of 250 to 300 seconds throughout the case. Because of multiple comorbidities such as chronic kidney disease and congestive heart failure, many patients need a staged approach—2 or more shorter procedures intervening on different segments or different limbs spanning a couple of weeks to months. Iodinated contrast agents and intravenous fluids need to be cautiously given, taking into consideration the Mehran score and the maximum allowable contrast dose for patients with chronic kidney disease^18,19^ or heart failure with presumed high-normal to high left ventricular end-diastolic pressure, using modified POSEIDON (Prevention of Contrast Renal Injury With Different Hydration Strategies) criteria to administer 1.5 mL/kg of body weight for 4 hours postintervention.^20^ Finally, a team-based approach that includes podiatrists; wound care specialists; interventional cardiologists trained to perform endovascular procedures; primary care doctors; physical therapists; endocrinologists for diabetes care; and plastic, orthopedic, and vascular surgeons is imperative to address the complexity of patients with CLI.

## CONCLUSION

This case demonstrates that the pedal-plantar loop technique may increase the success rate of revascularization of below-the-knee disease in patients with CLI.

## Notes

*This article meets the Accreditation Council for Graduate Medical Education and the American Board of Medical Specialties Maintenance of Certification competencies for Patient Care and Medical Knowledge.*

## References

[1] Schiavetta A, Maione C, Botti C, A phase II trial of autologous transplantation of bone marrow stem cells for critical limb ischemia: results of the Naples and Pietra Ligure Evaluation of Stem Cells study. Stem Cells Transl Med. 2012;1(7):572-578. doi: 10.5966/sctm.2012-0021 23197862 PMC3659723

[2] Fowkes FGR, Rudan D, Rudan I, Comparison of global estimates of prevalence and risk factors for peripheral artery disease in 2000 and 2010: a systematic review and analysis. Lancet. 2013;382(9901):1329-1340. doi: 10.1016/S0140-6736(13)61249-0 23915883

[3] Allie DE, Hebert CJ, Lirtzman MD, Critical limb ischemia: a global epidemic. A critical analysis of current treatment unmasks the clinical and economic costs of CLI. EuroIntervention. 2005;1(1):75-84. 19758881

[4] Thukkani AK, Kinlay S. Endovascular intervention for peripheral artery disease. Circ Res. 2015;116(9):1599-1613. doi: 10.1161/CIRCRESAHA.116.303503 25908731 PMC4504240

[5] Manzi M, Fusaro M, Ceccacci T, Erente G, Dalla Paola L, Brocco E. Clinical results of below-the knee intervention using pedal-plantar loop technique for the revascularization of foot arteries. J Cardiovasc Surg (Torino). 2009;50(3):331-337. 19543193

[6] Walker CM, Mustapha J, Zeller T, Tibiopedal access for crossing of infrainguinal artery occlusions: a prospective multicenter observational study. J Endovasc Ther. 2016;23(6):839-846. doi: 10.1177/1526602816664768 27558463 PMC5315197

[7] Rogers RK, Dattilo PB, Garcia JA, Tsai T, Casserly IP. Retrograde approach to recanalization of complex tibial disease. Catheter Cardiovasc Interv. 2011;77(6):915-925. doi: 10.1002/ccd.22796 20853359

[8] Montero-Baker M, Schmidt A, Bräunlich S, Retrograde approach for complex popliteal and tibioperoneal occlusions. J Endovasc Ther. 2008;15(5):594-604. doi: 10.1583/08-2440.1 18840044

[9] Nehler MR, Duval S, Diao L, Epidemiology of peripheral arterial disease and critical limb ischemia in an insured national population. J Vasc Surg. 2014;60(3):686-695.e2. doi: 10.1016/j.jvs.2014.03.290 24820900

[10] Teraa M, Conte MS, Moll FL, Verhaar MC. Critical limb ischemia: current trends and future directions. J Am Heart Assoc. 2016;5(2):e002938. doi: 10.1161/JAHA.115.002938 26908409 PMC4802465

[11] Mustapha JA, Saab F, McGoff T, Tibio-pedal arterial minimally invasive retrograde revascularization in patients with advanced peripheral vascular disease: the TAMI technique, original case series. Catheter Cardiovasc Interv. 2014;83(6):987-994. doi: 10.1002/ccd.25227 24214522

[12] Valle JA, Waldo SW. Worth an arm and a leg: the critical importance of limb ischemia. J Am Heart Assoc. 2018;7(16):e010093. doi: 10.1161/JAHA.118.010093 30369333 PMC6201406

[13] Bazan HA, Le L, Donovan M, Sidhom T, Smith TA, Sternbergh WC III. Retrograde pedal access for patients with critical limb ischemia. J Vasc Surg. 2014;60(2):375-382. doi: 10.1016/j.jvs.2014.02.038 24650744

[14] Sanghvi KA, Kusick J, Krathen C. Retrograde tibio-pedal access for revascularization of lower-extremity peripheral artery disease using a 6 Fr slender sheath: the “Pedal-First” pilot project. Cath Lab Digest. 2018;26(10). 30158324

[15] Manzi M, Palena LM. Treating calf and pedal vessel disease: the extremes of intervention. Semin Intervent Radiol. 2014;31(4):313-319. doi: 10.1055/s-0034-1393967 25435656 PMC4232433

[16] Gandini R, Del Giudice C, Simonetti G. Pedal and plantar loop angioplasty: technique and results. J Cardiovasc Surg (Torino). 2014;55(5):665-670. 24941239

[17] Troisi N, Turini F, Chisci E, Impact of pedal arch patency on tissue loss and time to healing in diabetic patients with foot wounds undergoing infrainguinal endovascular revascularization. Korean J Radiol. 2018;19(1):47-53. doi: 10.3348/kjr.2018.19.1.47 29353999 PMC5768506

[18] Aoun J, Nicolas D, Brown JR, Jaber BL. Maximum allowable contrast dose and prevention of acute kidney injury following cardiovascular procedures. Curr Opin Nephrol Hypertens. 2018;27(2):121-129. doi: 10.1097/MNH.0000000000000389 29261551 PMC6584943

[19] Faggioni M, Mehran R. Preventing contrast-induced renal failure: a guide. Interv Cardiol. 2016;11(2):98-104. doi: 10.15420/icr.2016:10:2 29588714 PMC5808627

[20] Brar SS, Aharonian V, Mansukhani P, Hemodynamic-guided fluid administration for the prevention of contrast-induced acute kidney injury: the POSEIDON randomised controlled trial. Lancet. 2014;383(9931):1814-1823. 24856027 10.1016/S0140-6736(14)60689-9

